# Application of microarray outlier detection methodology to psychiatric research

**DOI:** 10.1186/1471-244X-8-29

**Published:** 2008-04-23

**Authors:** Carl Ernst, Alexandre Bureau, Gustavo Turecki

**Affiliations:** 1 McGill Group for Suicide Studies, McGill University, Montreal, Canada; 2 Centre de recherche Université Laval Robert-Giffard and Department of social and preventive medicine, Université Laval, Canada; 3 Douglas Hospital Research Centre, Pavilion Frank B Common, Rm. F-3125, 6875 LaSalle, Blvd., Verdun, Montreal, Quebec, H4H 1R3, Canada

## Abstract

**Background:**

Most microarray data processing methods negate extreme expression values or alter them so that they do not lie outside the mean level of variation of the system. While microarrays generate a substantial amount of false positive and spurious results, some of the extreme expression values may be valid and could represent true biological findings.

**Methods:**

We propose a simple method to screen brain microarray data to detect individual differences across a psychiatric sample set. We demonstrate in two different samples how this method can be applied.

**Results:**

This method targets high-throughput technology to psychiatric research on a subject-specific basis.

**Conclusion:**

Assessing microarray data for both mean group effects and individual effects can lead to more robust findings in psychiatric genetics.

## Background

Currently used psychiatric nosology is based on a compilation of clinical symptoms into categories based primarily on symptom clustering and course. Diagnostic systems such as the current version of the DSM, allow for certain flexibility in the definition of diagnostic categories, with no assumption that each category of mental disorder is a completely discrete entity. As such, individuals diagnosed under a certain diagnostic class are not clinically homogeneous, there are no clear boundaries between classes, and different classes are not mutually exclusive. It is, therefore, unrealistic to expect that all subjects diagnosed with a given disorder will share a common psychopathological process, which would be associated with a common underlying biological process.

Most research efforts in psychiatry are directed towards the identification of group effects, negating the fact that significant etiological heterogeneity may exist. This limitation is particularly true for microarray research in psychiatry, where gene expression from different brain areas has been assessed comparing all affected subjects to non-affected subjects. A possible solution would be to carry out studies aiming at the identification of biologically meaningful effects focusing on single individuals or subgroups. This approach would mimic fruitful efforts in the identification of genetic factors underlying heterogeneous conditions such as, among others, spinocerebellar ataxia [1] and Alzheimer's disease [2].

Microarray data from psychiatric subjects can be investigated for individual or subgroup effects that may be of genuine biological significance. Specifically, we hypothesize that specific subgroups can be identified through microarray data screening for extreme expression values. Three previous studies have described how microarray data can be investigated on a subject-specific basis when analyzing data from cancer studies [3-5]. We suggest here that microarray experiments using brain-gene expression levels from psychiatric experiments (e.g. schizophrenia group vs non- schizophrenia group) can utilize microarray data not only for group mean effects (i.e. standard microarray analysis) but also, whenever possible, should evaluate expression levels by individual subjects.

Most microarray projects in psychiatry involve examining more than one neural region [6-9]. Specifically, researchers studying gene expression in brain tend to analyze more than one brain region on more than one array. This leaves researchers with gene expression data from multiple brain regions for each subject. This offers the possibility of using data from different arrays as confirmations of findings which may appear to be outliers. Any outlier on one chip that is also an outlier on a different chip may represent a valid finding.

Human brain has been categorized in two main ways: either by gross anatomical structure, the preference of imaging specialists, or by Brodmann region, the preference of neuro-anatomists. Irrespective of how the human brain is categorized anatomically, what is less obvious is whether gene expression varies between neighboring regions. Two recent, replicating studies suggest that brain gene expression of samples from the same individual, while non-identical, are biologically-related [9,10]. This sharing of similar expression patterns across samples allows for the exclusion of extreme values in the microarray data due to noise. This provides a potential to validate microarray data, particularly for variables that are extreme values, across chips. Those extreme values present across chips for the same probe set and the same individual may represent a true biological effect.

We have designed a method that can assess extreme values and utilizes expression data across chips from the same individual. The method will allow for the detection of any subjects that have probe set values that differ drastically from a mean and outside of a certain threshold (e.g. Standard deviations from a mean). This method, termed Extreme Values Analysis (EVA), takes into account the complex and heterogeneous nature of psychiatric diseases. We illustrate this approach in two different situations. First, in a publicly available sample where extreme values were simulated, and second, in a sample of subjects who died by suicide and sudden death controls. EVA functions to screen microarray data individual-by-individual in search for any extreme values that may signify some abnormality.

## Methods

We used a publicly available data set to first evaluate EVA. The data set comprises 9 subjects screened over 20 regions of the CNS and can be found here [9]. In this dataset, one of the authors (AB) inputted simulated extreme values for two subjects across all CNS regions for one randomly selected probe set each. The expression values were multiplied by 4^(1+0.25Z) ^for one of the probe sets and by 0.25^(1+0.25Z) ^for the other, where Z is a standard normal random variable which was generated independently for each CNS region. Another of the authors (CE), blinded to the experimental manipulation, applied the method to detect the inputted value(s). The rationale for this experiment is to determine if EVA can detect an artificially generated extreme value in one probe set from > 11 million different data points (10 subjects X 20 regions X~55,000 probe sets).

We assessed EVA in a second sample that comprised a group of suicide completers and sudden death controls. Information on the subjects, clinical variables, and microarray data quality of the suicide and sudden death controls can be found in Sequeira et al.,[6].

EVA can be applied under a control:experimental design (suicide and sudden death controls example) or in a one sample design (CNS screening example). We describe the control:experimental setting, although the description applies also to the one sample design. In the one sample design, all individual values are compared to the group to which they belong.

The mean and standard deviation (SD) of log_2_-transformed expression level is computed in the experimental group for all probe sets in every region. In our example, this was done in 2 cortical brain regions from suicide subjects. Log transformation stabilizes the variance, allowing comparison of SD across probe sets. After this step, the probe sets with the highest SD values were selected for further analysis. We used only those probe sets in the top 5% of SD values. We reasoned that these probe sets likely have individual values that are extreme, which accounts for a high SD value.

To buffer against detecting mathematical artifacts, EVA selects only those probe sets with high SD values in all regions. In our example, we selected probe sets that were common across both cortical regions. Next, we assess whether the same subject is responsible for the high SD value across brain regions. We set as criterion for an extreme expression a value of ± 3 fold greater than the mean expression level of the specific probe set among the control group (in our example, the sudden death controls). This approach operates on the assumption that neighboring brain regions are not discrete units and that gene expression should not vary widely from one cortical region to another. Even if brain region-specific expression is more common, it is not expected that a subject that is an outlier in one region is necessarily an outlier in a neighboring region. In other words, extreme values that are detected across multiple brain regions are more likely to represent real biological phenomena. We note that this method is conservative.

Individual expression values also have to be outside of 1.5 SD's of the control group, after having met the above criteria. While we selected 1.5 SD's from the mean of the opposite group, this number can be changed depending on the false discovery level acceptable to the experimenter. Manipulating the SD threshold establishes the false discovery rate (FDR) of the experiment.

The statistical significance of each identified outlier can be assessed by computing the p-value of the subject's expression values for a probe set in the multiple brain regions compared to the multivariate distribution of the expression values in the control group. The null distribution of the log_2_-transformed probe set-specific expression is estimated by fitting a normal mixed model where the subject effect is random. Letting, X_ij _be the probe set-specific expression of the i^th ^subject in the j^th ^brain region, and Y_ij _= log_2_(X_ij_), this model has the form:

Y_ij _= μ_j _+ a_i _+ e_ij_, a_i_~N(0,τ^2^), e_ij_~N(0,σ^2^)

where μ_j _is the region-specific mean expression, a_i _is the subject random effect and e_ij _is the residual. We fit such a model by restricted maximum likelihood (REML) using the maanova package [11] for the R statistical software [12]. The subject random effect captures the expected correlation between expression in different brain regions of the same subject. The p-value for the observed deviation of the log_2_-transformed expression level of the i^th ^subject from the mean of the group of reference ${\hat{\mu }}_{\text{j}}$, j = 1,..., J (or observed fold change on the original scale) is given by

$$\text{P}\left(\left|{\text{Y}}_{1}-{\hat{\mu }}_{1}|>|{\text{y}}_{\text{i}1}-{\hat{\mu }}_{1}|,\ldots ,|{\text{Y}}_{\text{J}}-{\hat{\mu }}_{\text{J}}|>|{\text{y}}_{\text{iJ}}-{\hat{\mu }}_{\text{J}}\right|\right)$$

which we compute using a multivariate t-distribution with the covariance matrix estimated under the normal mixed model.

## Results

### EVA in partially simulated data

We tested EVA in a sample data set that included 20 different CNS regions [9]. This dataset was selected because A) we could test how the method works with the RMA algorithm and B) we could demonstrate the method in a one-sample case.

**Figure 1:**
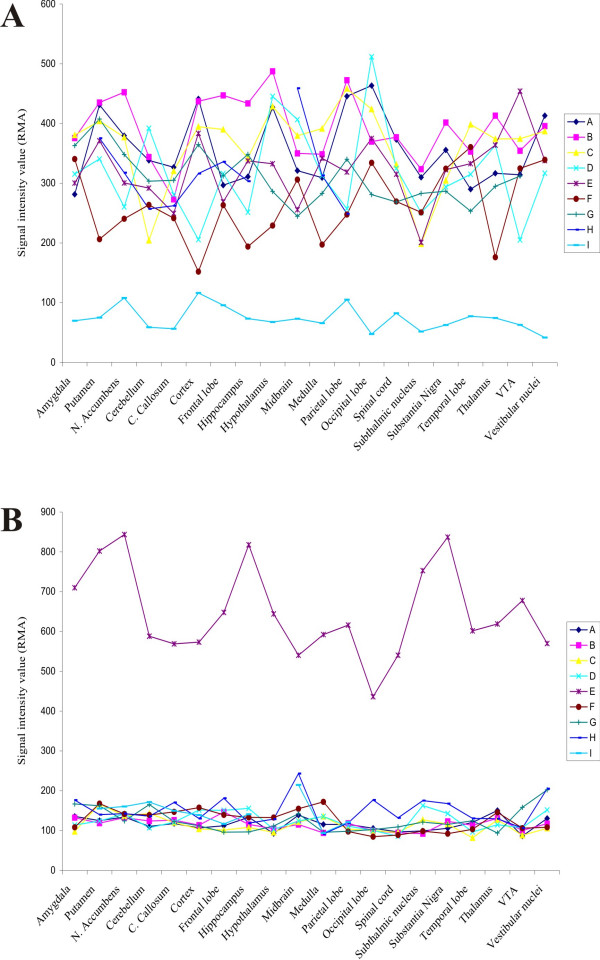
**Microarray expression values demonstrating two subjects who passed all EVA criteria in a one sample case**. A) Extreme low expressor (light blue trace) compared to other subjects in the same sample for one probe set identified across multiple CNS regions. Each subject is represented by a letter (A, B, C...). B) Extreme high expressor (purple trace) compared to other subjects in the same sample for one probe set across multiple CNS regions.

We began by computing the standard deviation (SD) for three of the 20 CNS regions described in this data set. The probe sets in the top 5% of SD values was selected for each of three regions and those probe sets that were common to all regions were selected. Five hundred forty-five probe sets were common to all three regions. Next, we screened for any individual values that lay outside of ± 1.5 SD's and was three-fold different from the mean. There were 14 genes that were found to be 3-fold greater than the mean and outside of +1.5SD's and 245 values that were three fold below the mean and outside of -1.5SDs. Each of these values was then cross-referenced across all 20 CNS regions. Two probe sets were found that met all criteria (1 above the mean for one subject and one below the mean for another subject). These were the probe sets that had been artificially altered (Figure 1).

### EVA in real microarray data

To demonstrate this technique, we used a sample that included 6 control subjects and 8 suicide completers with microarray data from BA 8/9 and BA 11. We first screened all expression values for MAS 5.0 present/absent call leaving 14,896 probe sets in BA 8/9 and 14,412 probe sets in BA 11. We next calculated a standard deviation for all probe sets from suicide subjects. This was done using log_2_-transformed expression values. We then selected the probe sets with the highest SD values (top 5%) from both BA 8/9 and BA11.

Any probe sets that was identical to both BA 8/9 and BA 11 after SD filtering was selected. There were 180 probe sets that were common to both regions. Next, to account for the variability of expression in control values, we searched the data for any suicide data point greater than 3-fold from the control mean and outside of 1.5 SD's. We reasoned that an extreme value across all regions for the same subject(s) could represent a biologically relevant event.

Beginning in BA 8/9, we filtered the 180 probe sets for those probe sets from suicide completers outside of 3-fold from the control mean. There were 20 probe sets where X_ij _(a particular expression value from a particular subject in a given brain region) was not outside of 3-fold from the control mean. From the 160 remaining probe sets, 108 probe sets were also outside of 1.5 SD's in BA 8/9. Probe sets from BA 11 were then filtered for these probe sets.

Table 1 lists the probe sets across the eight suicide completers and the individual p-values associated with each subject. From 108 probe sets that passed all EVA criteria in BA 8/9, 69 passed all EVA criteria in BA 11. Included in this list of probe sets are a number of genes that have been linked to suicide before including the FGF family [13], NTRK2 [14], and members of the ubiquitin family [15]. Of note, from the table, is that for a number of probe sets there is more than one subject who has an extreme expression value reaching a significance level.

**Table 1:** HG-U133 plus 2 probe sets that met all EVA criteria. Numbers represent p-values generated for each probe set across each subject (S). Subjects who met EVA criteria have p-values underlined. Note that p-values are generated from the number of SD's from the mean, therefore some subjects with very small p-values may be outside of a given number of SD's but < 3-fold different than the mean.

Probe set	S1	S2	S3	S4	S5	S6	S7	S8
233814_at	0.00011	0.262901	0.060973	0.119425	0.340339	0.065993	0.291037	0.044823
225440_at	0.003777	0.041856	0.549775	0.617546	0.000754	0.044895	0.52789	0.000355
203638_s_at	0.008775	0.076607	0.099598	0.154338	0.000159	0.089298	0.01575	0.357839
214680_at	2.89E-05	0.010459	0.007155	0.001488	0.00034	0.007818	0.000768	0.148031
37170_at	0.001734	0.002016	0.095708	0.010663	0.000198	0.000259	0.003458	0.140702
227556_at	0.00074	0.397533	0.000597	0.010661	0.291994	7.80E-07	0.100161	0.010813
229917_at	0.00895	0.170125	0.421083	0.044559	0.401754	0.000248	0.055834	0.003505
227330_x_at	0.018916	0.412934	0.378829	0.053889	0.002547	0.576196	0.037467	0.46594
231804_at	0.411319	0.07522	0.566597	0.115634	0.485137	0.489949	0.28554	0.012676
200904_at	0.066736	0.080689	0.236431	0.504115	0.009175	0.255437	0.466512	0.105319
230141_at	0.041214	0.157873	0.408831	0.09835	0.013597	0.067947	0.333108	0.004167
222020_s_at	6.92E-05	0.071294	0.239087	0.018151	0.271669	0.043433	0.044077	0.428814
213812_s_at	0.004793	0.002408	0.151259	0.121657	0.15422	0.030286	0.022571	0.02784
201505_at	0.316616	0.570798	0.160905	0.60401	0.000337	0.19324	0.157064	0.00128
240467_at	0.103838	0.007113	0.076142	0.001703	0.050236	0.332126	0.148029	0.199109
229861_at	0.005266	0.047216	0.055746	0.000202	0.028745	0.028233	0.019457	0.00037
225872_at	0.001483	0.528714	0.628325	0.427906	0.000266	0.396505	0.019428	0.194352
241758_at	0.625246	0.629225	0.435153	0.452194	0.005473	0.282962	0.223662	0.001718
214449_s_at	0.400041	0.272717	0.30737	0.020469	0.000229	0.040009	0.340049	0.000813
200648_s_at	0.011284	0.10939	0.371707	0.02763	0.013746	0.07564	0.02506	0.264293
221795_at	0.000617	0.006302	0.070084	0.008107	0.005545	0.039509	0.01825	0.284639
204379_s_at	0.046084	0.083475	0.391619	0.237209	0.025689	0.079905	0.073448	0.22916
215172_at	0.109908	0.139031	0.199485	0.152187	0.155703	0.203207	0.144057	0.001802
203324_s_at	0.005691	0.092966	0.150765	0.000126	0.00021	0.056637	0.205541	0.183977
202800__at	0.077088	0.491247	0.411825	0.162812	0.013449	0.102756	0.028244	0.452363
236223_s_at	0.030208	0.196886	0.209618	0.000686	0.25837	0.13088	0.373756	0.268783
209023_s_at	5.10E-05	0.000708	0.030401	0.001934	0.251739	0.002706	2.10E-05	0.054205
213593_s_at	0.005575	0.001263	0.108932	0.005416	0.007774	0.20946	0.083709	0.036244
222249_at	0.101364	0.131587	0.040535	0.007024	0.009912	0.096597	0.00014	0.012586
220460_at	0.018979	0.539318	0.195645	0.120094	0.009469	0.067115	0.013541	0.346847
201656_at	0.000481	0.13031	0.064596	0.010041	0.000455	0.018925	0.14539	0.117989
235775_at	0.000373	0.079169	0.040214	0.11224	0.000676	0.222721	0.208163	0.049906
204516_at	9.40E-05	0.051213	0.096458	0.027735	0.003274	0.277745	0.208683	0.17238
201843_s_at	0.047846	0.096131	0.228376	0.016614	0.010238	0.104263	0.005888	0.155651
204712_at	0.024623	0.417385	0.556647	0.127233	0.010306	0.182687	0.121824	0.39341
224736_at	0.002159	0.02277	0.118888	0.114733	0.126571	0.333625	0.187404	0.021966
214203_s_at	0.018997	0.038021	0.062828	0.074293	0.00133	0.017613	0.003911	0.047244
200914_x_at	0.010589	0.005141	0.075284	0.058115	0.042784	0.274358	0.103149	0.103501
222404_x_at	0.003613	0.040237	0.059019	0.220239	0.151453	0.188272	0.358947	0.103866
229553_at	0.008515	0.022552	0.02734	0.070236	0.14418	0.145378	0.331622	0.223119
203249_at	0.139981	0.138389	0.229283	0.100477	0.004921	0.094854	0.07573	0.002453
203041_s_at	0.01742	0.111182	0.227792	0.402694	0.014214	0.310238	0.14055	0.174457
209292_at	0.068175	0.345992	0.507253	0.01934	0.006611	0.099673	0.002438	0.275859
226084_at	0.00373	0.005272	0.059754	0.096792	0.076702	0.642227	0.101137	0.153124
204976_s_at	0.00165	0.083667	0.100336	0.152992	0.00468	0.159244	0.449992	0.583733
212368_at	0.013283	0.016864	0.07334	0.086183	0.13413	0.246171	0.126799	0.293217
211962_s_at	0.00228	0.17037	0.178376	0.026324	0.0011	0.017557	0.011854	0.278736
226228_at	0.090147	0.423425	0.572415	0.125902	0.015242	0.100574	0.077677	0.633229
213954_at	0.002753	0.001918	0.022841	0.013454	8.47E-05	0.018911	0.075725	0.077512
213922_at	0.004719	0.003408	0.109555	0.024766	0.07629	0.417034	0.042621	0.199903
221517_s_at	0.001167	0.00413	0.048293	0.022654	0.003271	0.150882	0.080039	0.011072
227099_s_at	0.047549	0.092241	0.069821	0.333917	0.000425	0.00361	0.200933	0.009189
201737_s_at	0.000159	0.012972	0.219393	0.110936	0.092836	0.273891	0.078928	0.035264
214279_s_at	0.008594	0.363441	0.365898	0.01142	0.036218	0.014181	0.059127	0.066575
205709_s_at	0.007059	0.021998	0.110402	0.359529	0.08021	0.218438	0.306235	0.206374
225810_at	0.039054	0.256396	0.159142	0.452173	0.001525	0.010676	0.017581	0.130546
226435_at	0.150996	0.248593	0.432525	0.004589	0.032368	0.12243	0.072016	0.216067
226364_at	0.310524	0.026062	0.011704	0.02649	0.026889	0.178983	0.048683	0.005222
240482_at	0.318335	0.475263	0.195512	0.214718	0.151663	0.390872	0.003578	0.001316
204881_s_at	0.00017	0.068969	0.075294	0.117202	0.020759	0.039673	0.310769	0.084319
203841_x_at	0.000201	0.005823	0.089297	0.006843	0.052791	0.114169	0.037911	0.052824
212677_s_at	0.021412	0.003193	0.033988	0.022312	0.03778	0.128097	0.090665	0.060285
201502_s_at	0.001372	0.006379	0.167335	0.041531	0.035423	0.070657	0.263158	0.614172
240299_at	0.017658	0.276529	0.197699	0.008206	0.0622	0.215909	0.087937	0.025787
212423_at	0.024073	0.015847	0.140173	0.140948	0.19045	0.020469	0.19758	0.399876
228811_at	0.290584	0.349143	0.140068	0.371578	0.50686	0.002176	0.066324	0.002291
224737_x_at	0.000367	0.288851	0.112457	0.002641	0.097181	0.24341	0.219535	0.021662
201019_s_at	0.003446	0.00455	0.276132	0.181832	0.064501	0.680406	0.179361	0.099558
229281_at	0.090713	0.333165	0.42729	0.241316	0.000732	0.019187	0.025054	0.314121

## Discussion

The extreme values analysis, or EVA, is a method to detect individual or subsets of outliers for a given probe set in microarray experiments. The rationale for this type of experiment is that psychopathology is not necessarily group specific but more likely sub-group or subject specific. The method outlined here uses log-transformed data to determine which probe sets have the highest variance and screens out those probe sets with little variation. This step is intended to select those probe sets with values that deviate widely from the mean. Next the method compares individual data points to a control mean, and searches for any 3-fold changes. Selected values also have to be outside of 1.5 SD's from the mean. These values were considered extreme expression values. These extreme expression values were next verified in one other cortical region to determine if they were extreme expression values in other cortical regions as well. We reasoned that the use of other cortical regions functioned as replicate experiments and enforced the finding.

We also evaluated this method in a one sample case after inputting artificial values for one probe set across all CNS regions in RMA data. EVA was able to detect the inputted value; the only difference between the control:experimental case and one sample case is the mean value used: In the one sample case the mean used includes the extreme value while in the control:experimental case it does not.

The use of multiple cortical regions as within-subject replicates is a way to detect true extreme expression values in individual subjects. Operating under the assumption the gene expression in one cortical region is generally similar in neighboring cortical regions, we propose that different chips for the same subject can be used as replicate experiments, if probe set outliers on an individual specific basis are being investigated. If an observed outlier is a real biological event, it is very probable that the same subject on the same probe set will also be an outlier in a neighboring region. Consider, for example, the family with a deletion in the MAOA gene [16]. Had this family undergone post-mortem microarray analysis as a part of a larger sample of subjects, EVA would have detected the MAOA decrease in expression whereas microarray analysis using mean group effects would not have. Using multiple brain regions as replicates does undermine the idea that gene expression is different across different brain regions, which it is [10,17]; however, it means that if an effect is detected, it is likely real and robust.

### Comparison to PPST method

The PPST method [5] counts the number of subjects in both control and experimental group outside of the 95^th ^percentile of the opposite group. The FDR is therefore controlled by altering the percentile threshold. EVA uses the SD from the opposite group and counts the number of subjects that are outside a given SD value (± 1.5 SDs in this study). Selecting more stringent SD values allows for direct manipulation of the FDR. In this study a liberal cutoff was chosen (outside of 1.5SD's). The FDR among the detected outliers could be estimated from the p-value of the subject's expression values using standard methods such as that of Reiner et al. [18]

### Comparison to COPA method

Cancer outlier profile analysis (COPA) is another outlier detection that has proved fruitful in the past[4]. This technique normalizes all probe sets (one sample design) and calculates the 75^th^, 90^th^, and 95^th ^percentiles for each probe set and rank-orders them by percentile score. A prioritized list of probe set with some subjects that have extreme expression values is then investigated. Tibshirani and Hastie [3] introduce the outlier-sum statistic in their paper to improve on the COPA method. Their method differs from COPA by the standardization procedure of each probe set expression level using the median and median absolute deviation.

There are some caveats to be aware of before proceeding with this approach to screen microarray data. Firstly, the method is very conservative and likely has a high beta error rate. It is very likely that there were a number of true positives that were not detected because of the rigidity of the design. Some parameters may need to be adjusted to allow more probe sets to pass filtering (e.g. top 10% of SD values instead of the top 5% being used). Second, this method has the disadvantage of requiring a number of replicates per individual, a component that could be cost-prohibitive. Third, the method can only be used to study genes whose expression levels are similar across brain regions. Finally, we note that all probe-level microarray algorithms dampen extreme values at the scanner. This method is conservative and could only be used to investigate extreme values after initial processing.

Our view for this technique is as another analysis technique to further explore microarray data, in conjunction with more mainstream techniques [19]. This method, termed Extreme Values Analysis, can detect extreme differences in gene expression on a subject-by-subject basis from microarray data across different chips. The method uses high-throughput technology in a non-biased way to understand psychiatric disease for each subject investigated.

## Competing interests

The author(s) declare that they have no competing interests.

## Authors' contributions

CE conceived of the study, wrote the manuscript, and analyzed data. AB designed the statistical test and wrote the manuscript. GT participated in study design and coordination, and wrote the manuscript. All authors read and approved the final manuscript.

## Pre-publication history

The pre-publication history for this paper can be accessed here:


